# Insights on the Bonding Mechanism, Electronic and Optical Properties of Diamond Nanothread–Polymer and Cement–Boron Nitride Nanotube Composites

**DOI:** 10.3390/molecules29194693

**Published:** 2024-10-03

**Authors:** Diamond C. Domato, Art Anthony Z. Munio, Naomi Jane P. Jacosalem, Dexter Rhys T. Fuentes, Leo Cristobal C. Ambolode

**Affiliations:** 1 Department of Physics, Mindanao State University–Iligan Institute of Technology, Iligan City 9200, Philippines; naomijane.jacosalem@g.msuiit.edu.ph (N.J.P.J.);; 2 Center for Nanoscience Research, Premier Research Institute of Science and Mathematics (PRISM), Mindanao State University–Iligan Institute of Technology, Iligan City 9200, Philippines; dexterrhys.fuentes@g.msuiit.edu.ph; 3 College of Arts and Sciences, Jose Rizal Memorial State University, Tampilisan 7116, Philippines

**Keywords:** diamond nanothread, polymer, cellulose, epoxy, boron nitride nanotube, calcium silicate hydrate, cement composite, nanocomposite, density functional theory

## Abstract

The success of composite materials is attributed to the nature of bonding at the nanoscale and the resulting structure-related properties. This study reports on the interaction, electronic, and optical properties of diamond nanothread/polymers (cellulose and epoxy) and boron nitride nanotube/calcium silicate hydrate composites using density functional theory modeling. Our findings indicate that the interaction between the nanothread and polymer is due to van der Waals-type bonding. Minor modifications in the electronic structures and absorption spectra are noticed. Conversely, the boron nitride nanotube–calcium silicate hydrate composite displays an electron-shared type of interaction. The electronic structure and optical absorption spectra of the diamond nanothread and boron nitride nanotube in all configurations studied in the aforementioned composite systems are well maintained. Our findings offer an electronic-level perspective into the bonding characteristics and electronic–optical properties of diamond nanothread/polymer and boron nitride nanotube/calcium silicate hydrate composites for developing next-generation materials.

## 1. Introduction

To advance existing technology usually boils down to engineering materials at the nanoscale [1]. Incorporating advanced materials into traditional materials, such as nanomaterials, and integrating them into polymer and ceramic matrices can significantly improve the resulting mechanical properties and introduce new functionalities to the composites [2,3]. The potential applications of composites are in diverse fields, ranging from aerospace to construction materials [4,5,6]. Among the list of composites, polymer-based and cement-based composites are the most influential and have great economic value due to their high volume of utilization.

Polymer composites with carbon-based nanomaterials (CNMs) have been a subject of interest due to the remarkable ability of CNMs like carbon nanotubes (CNTs) and graphene to reinforce, significantly improving mechanical properties and adding new functionalities to polymer composites [7,8,9,10,11,12]. For instance, an investigation of the mechanical properties of CNT-reinforced polymer composites using a closed-form micromechanical interphase model considering the diameter of the CNT, the thickness of the interphase, and the mechanical properties of the CNT and polymer revealed that the elastic modulus of the CNT/polymer composite increases by up to 108% with the addition of the CNT [13]. However, despite these benefits, poor interface adherence is still one of the interface-level issues that CNMs in polymer composites confront. Research is increasingly focusing on addressing this problem to maximize the reinforcing efficiency of CNMs in polymer composites, and this includes looking into substitute reinforcements with better interfacial interaction.

Recently, a new class of carbon nanomaterial known as the diamond nanothread (DNT) has been gaining attention due to its unique geometrical structure and outstanding properties. This nanomaterial is typically produced by controlled compression/decompression of aromatics such as benzene, aniline, pyridine, thiophene, furan, etc. [14,15,16,17,18,19,20,21,22,23,24,25,26,27,28]. A DNT consists of a one-dimensional chain of sp^3^-bonded carbon atoms, which can be arranged in several ways [29,30,31], with their outer surface functionalized by hydrogen atoms. This bonding pattern of DNT produces a robust structure with an ultralight density. DNTs have been reported to possess an ideal strength of 26.4 nN (134 GPa), Young’s modulus of 850 GPa, specific strength of $2.6\times 10^{7}$ N∙m/kg, and specific stiffness of $2.8\times 10^{8}$ N∙m/kg [32]. These outstanding mechanical properties of DNTs make them ideal reinforcements for nanocomposites [33,34,35,36]. Previous reports have demonstrated that DNTs even outperform CNTs as a reinforcing material for enhancing the thermal-mechanical properties of polymer composites [33,35,36]. Zhan et al. reported that DNT demonstrates remarkable torsional stiffness that is nearly three times greater than that of CNT and that the interfacial shear strength (ISS) in DNT bundles surpasses that observed in CNT bundles [37]. Further study revealed that the ISS at the DNT-polyethylene (PE) interface is comparable to that observed in the CNT-PE interface [33]. Additionally, a simulation study on the mechanical properties of bulk poly (methyl methacrylate) (PMMA), CNT/PMMA composite, and DNT/PMMA composite under uniaxial tension reveal that the Young’s modulus and yield stress of the DNT/PMMA composite are 85% and 15% higher than those of the PMMA matrix [34]. 

While the mechanical properties of DNT and its effectiveness as a reinforcement in polymer composites have been investigated, the electronic behavior of DNT with polymer composites has not been elucidated. Exploring the electronic properties of DNT combined with polymer matrices (i.e., biopolymers, epoxies, thermoplastics, etc.) may provide insights into their interaction and offer alternative means to design high-strength polymer composites. Among these polymers, cellulose is the most abundant natural polymer on earth; it has the molecular formula ${\left(C_{6}H_{10}O_{5}\right)}_{n}$ and is known for its hydrophilicity, biodegradability, biocompatibility, nontoxicity, low cost, etc. [38,39]. Studies on cellulose with CNMs have been actively conducted and demonstrated enhancement of composite properties such as Young’s modulus, tensile strength, adsorption rate, porosity, etc. [40,41]. In addition to cellulose, epoxy, one of the most widely used polymer resins, has become widely utilized in the development of polymer composites due to its exceptional properties including excellent chemical and corrosion resistance, high adhesion strength, and good thermal, electrical, and adhesive properties [42]. Several reports on CNMs/epoxy combined material have revealed improved thermal, electrical, and mechanical properties [43,44,45,46,47,48,49,50,51]. For these reasons, delving into the interaction of cellulose and epoxy with DNT is also noteworthy.

Another research field of interest is smart-cement-based composites, which show promise in revolutionizing the construction industry. Unlike conventional cement-based composites, smart cement incorporates advanced nanomaterials to improve its mechanical properties and add functionalities such as piezoelectric, thermoelectric, self-cleaning, self-sensing, and electromagnetic interference shielding properties [52,53,54,55]. This emerging technology has the potential to enhance the durability, safety, and sustainability of infrastructure projects, making it a promising solution for the challenges of modern construction and the environment as a whole. 

In the recent past, graphene, CNTs, metal oxide nanoparticles, and polymers have been the topic of nanoreinforcement in cement-based composites [56,57,58]. For instance, combining a small percentage of graphene oxide into a calcium silicate hydrate (C-S-H) matrix enhances the flexural and compressive strength by 50% and 28% [59]. Several papers have obtained similar conclusions, which are communicated in the following works [60,61,62,63]. The improvement in the macroscopic mechanical properties is attributed to the nanomaterials, which served as a seeding nanomaterial for the growth of C-S-H, thus producing more C-S-H in the composite [64]. A similar trend is observed in the mechanical performance of a cement and CNT composite [65]. Carbon nanostructures as filler materials reduced the composite’s porosity, thus shielding it from the penetration of chloride ions [66]. The application of nanotechnology in cement composites holds great promise as the building material for smart cities. However, the diversity of nanomaterials for cement-based composites makes the study challenging. Currently, most research is focused on graphene-based nanostructures, with minimal attention paid to boron nitride nanomaterials, which perform similarly to graphene-based nanomaterials. 

Boron nitride nanomaterials are remarkable nanomaterials with a wide range of applications, including their potential use in cement composites [67,68,69,70]. These nanotubes are structurally similar to carbon nanotubes but comprise boron and nitrogen atoms. The mechanical properties of the boron nitride sheet are also comparable with that of carbon-based nanomaterials, with a Young’s modulus and fracture strength of 0.865 TPa and 70.5 Gpa, respectively [71]. Meanwhile, BNNT has a Young’s modulus and tensile strength of 1.3 GPa and 33 GPa, respectively [72]. Its incorporation into the C-S-H matrix at the electronic level has not yet been investigated. This information is crucial for understanding the bonding characteristics of the BNNT and C-S-H layers.

This work examines the bonding mechanism, electronic structure, and optical properties of DNT/cellulose, DNT/epoxy, and BNNT/C-S-H nanocomposites using density functional theory (DFT) calculations. The results indicate weak interactions between the DNT and polymers with minimal charge transfer. Thus, only minor alterations in the electronic and optical properties of the nanothread are observed. On the one hand, the C-S-H/BNNT composite demonstrates bonding with electrons overlapping between the BNNT and the prototype C-S-H. The wide bandgap of the BNNT is well maintained in the composite structure, as well as the electronic and optical properties. The results presented here will serve as a reference for developing advanced polymer-based composites and cement-based composites.

## 2. Results and Discussion

In this section, we present the atomic configurations, electronic band structures and density of states, and optical absorption spectra of DNT/cellulose, DNT/epoxy, and C-S-H/BNNT composites.

### 2.1. DNT/Cellulose and DNT/Epoxy Nanocomposites

**Figure 1:**
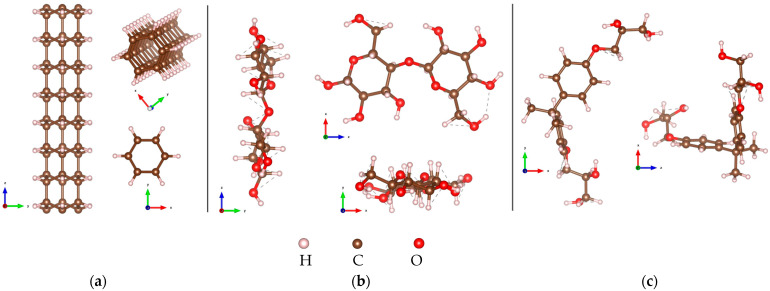
Atomic configurations of: (**a**) tube (3,0) diamond nanothread; (**b**) cellulose chain; (**c**) epoxy.

The optimized atomic configurations of the sp^3^ tube (3,0) DNT, cellulose chain, and epoxy are illustrated in Figure 1. The DNT shown in Figure 1a consists of a total of 96 atoms, which can be viewed as stacked benzene rings forming a one-dimensional thread. Each benzene ring has six sp^2^ hybridized carbon atoms that will convert to covalently tetrahedral sp^3^ bonding carbon atoms in the nanothread through adjacent interconnection [29]. The C-H bond length is measured to be 1.10 Å. The C-C bond length between the carbon atoms in a benzene ring is 1.54 Å, while those between the adjacent benzene rings are 1.58 Å. These measured C-C bond lengths for the DNT are in good agreement with the computed values (1.54–1.57 Å) from the previous report [29]. Additionally, the bond angles within the hexagonal benzene rings range from 106.4° to 106.5°. The modeled cellulose chain has a total of 45 atoms, as presented in Figure 1b. Its C-C, C-H, and C-O bond lengths range from 1.52 to 1.54 Å, 1.10 to 1.11 Å, and 1.40 to 1.43 Å, respectively, while the O-H bond has a length of ~ 0.97 Å. The bond angles within the chain rings are measured to be 108.4° to 111.7°. In Figure 1c, the selected epoxy model is illustrated and consists of a total of 55 atoms. The C-C, C-H, C-O, and O-H bond lengths range from 1.39 to 1.54 Å, 1.10 to 1.11 Å, 1.37 to 1.43 Å, and 0.96 to 0.97 Å, respectively. The bond angles within the rings are also obtained to be 117.1–122.2°.

When modeling the composites, the aim is to represent the complex interactions and properties of these materials as accurately as possible while maintaining computational feasibility. In real-world systems, carbon nanomaterials (e.g., carbon nanotubes) are often dispersed within the polymer matrix. Herein, these simulations model the interface behavior and the influence of the orientation of nanomaterials. These are particularly useful for composites where the distribution and orientation of nanomaterials can vary, providing insights into their bonding mechanisms and properties.

**Figure 2:**
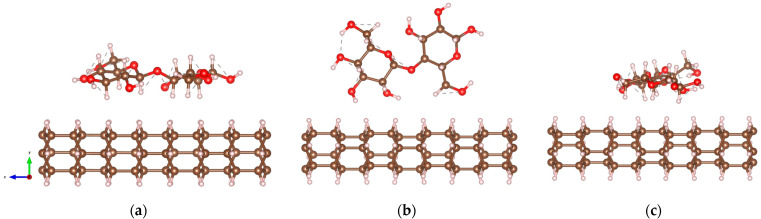
Optimized structures of DNT/cellulose composites with varying orientations of cellulose on the nanothread, labeled as: (**a**) DNT/cellulose—1; (**b**) DNT/cellulose—2; (**c**) DNT/cellulose—3. The carbon, hydrogen, and oxygen atoms are represented by brown, off-white, and red spheres, respectively.

Figure 2 displays the optimized structures of DNT/cellulose composites. The minimum interaction distances $d_{min}(Å)$ between DNT and cellulose, calculated binding energies $E_{b}$, and the results of Bader charge transfer analyses ∆ρ are obtained and summarized in Table 1. The first configuration illustrated in Figure 2a, where the backbone of the cellulose lies parallel to the nanothread, yields the strongest interaction relative to the interaction energies of the DNT/cellulose complex systems. This is attributed to the orientation of cellulose on the DNT, which leads to a more expansive interface. The shortest distance between the non-hydrogen atoms of the nanothread and cellulose is 3.67 Å (C-O), and the calculated binding energy is −0.797 eV. In Figure 2b, the cellulose is oriented perpendicular to the tube, the minimum distance between their non-hydrogen atoms is measured to be 3.42 Å (C-O), and the binding energy is −0.730 eV. In Figure 2c, where the cellulose is projected along its *z*-axis (as illustrated in Figure 1b), such that the cellulose in Figure 2a is rotated by 90 degrees, the minimum distance and binding energy are calculated to be 3.48 Å (C-O) and −0.726 eV, respectively.

**Figure 3:**
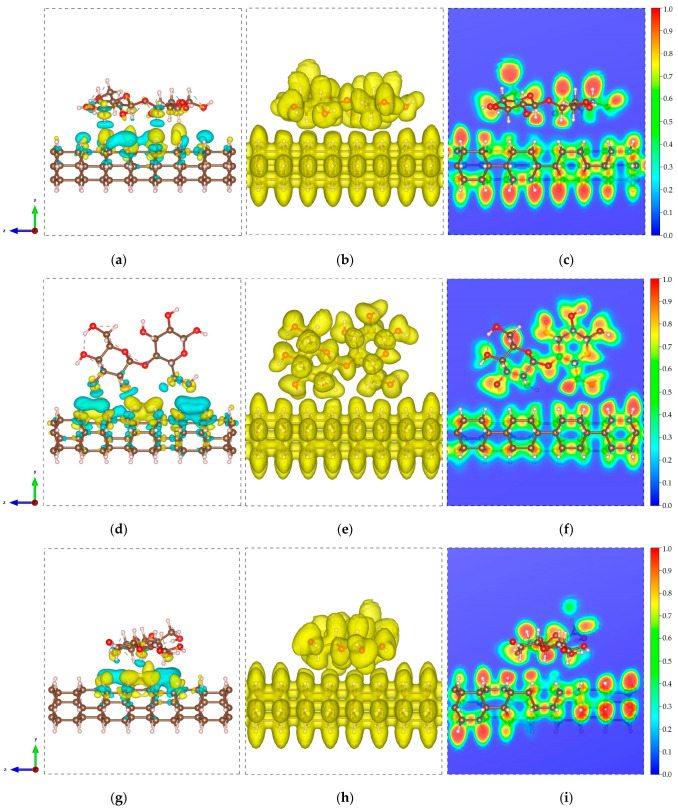
Electronic visualization of the DNT/cellulose composites: (**a**,**d**,**g**) charge density difference (CDD) (0.0002 e/Å^3^); (**b**,**e**,**h**) ELF isosurface (0.4); (**c**,**f**,**i**) 2D ELF contour of (**a**–**c**) DNT/cellulose—1, (**d**–**f**) DNT/cellulose—2, and (**g**–**i**) DNT/cellulose—3. The yellow and cyan isosurfaces denote the accumulation and depletion of the electronic charge, respectively.

In all structures, the C-C and C-H bond lengths of the DNTs are measured to be 1.54–1.58 Å and 1.10 Å, while the bond angles within the benzene rings are measured to be 106.3–106.5°. Similarly, the C-C, C-H, C-O, and O-H bond lengths of the cellulose have values of 1.52–1.54 Å, 1.10–1.11 Å, 1.40–1.43 Å, and 0.97 Å, respectively, with measured bond angles of 108.3–111.9°. These results indicate minimal changes in the structures of the subsystems in the composites, which imply weak interaction between the nanothread and cellulose. Furthermore, the negative values of the binding energies indicate an exothermic process. These non-bonded interactions between the DNT and cellulose and the obtained values of $d_{min}$ suggest a physical type of adsorption dominated by vdW forces. To further examine these interactions, the CDD and ELF are obtained for all structures and presented in Figure 3. The CDD is calculated by taking the difference between the charge density of the composites and the charge densities of the isolated constituents. The CDD isosurfaces presented in Figure 3a,d,g show that the charges are mainly distributed along the nanothread interface. The yellow and cyan isosurfaces imply gain and loss of charge, respectively.

To inspect charge transfer, a Bader charge transfer analysis is performed. A 0.008 e (DNT/cellulose—1) and 0.006 e (DNT/cellulose—3) charge transfer from the cellulose to the DNT are acquired. In contrast, a 0.004 e charge transfer from the DNT to cellulose is obtained for the DNT/cellulose—2 composite. The ELF isosurface and 2D ELF contour are displayed in Figure 3b,c,e,f,h,i. ELF [73] is a measurement of electron localization in atomic and molecular systems and is represented on a scale from 0 to 1. When ELF values exceed 0.7, electrons are considered localized, indicating the presence of core or bonding regions or lone pairs. Meanwhile, when ELF values fall between 0.7 and 0.2, electron localization resembles that of an electron gas and is typical of metallic bonds [74]. The ELF isosurface illustrated in Figure 3b,e,h shows a relatively uniform electron density across the composites. The 2D ELF contour displayed in Figure 3c,f,i further confirms the non-bonding interactions between cellulose and DNT. No overlapping of ELF is observed, as evident in the distinct blue region between the cellulose and nanothreads. These ELF diagrams provide additional confirmation of the noncovalent interaction between the nanothread and cellulose.

**Figure 4:**
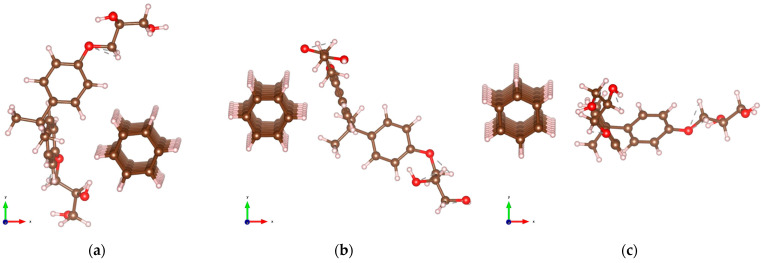
Optimized structures of DNT/epoxy composites with varying orientations of epoxy on the nanothread: (**a**) DNT/epoxy—1; (**b**) DNT/epoxy—2; (**c**) DNT/epoxy—3. The carbon, hydrogen, and oxygen atoms are represented by brown, off-white, and red spheres, respectively.

The optimized atomic configurations of DNT/epoxy composites are displayed in Figure 4. The calculated minimum interaction distance $d_{min}$, binding energy $E_{b}$, and charge transfer ∆ρ are summarized in Table 2. The first configuration illustrated in Figure 4a (DNT/epoxy—1), where the epoxy wraps around the thread, has −0.224 eV binding energy and the minimum distance between the non-hydrogen atoms is measured to be 3.56 Å (C-O). In the second configuration (DNT/epoxy—2), where the backbone of the epoxy is oriented slightly perpendicular to the thread, as displayed in Figure 4b, the closest distance between their atoms is 3.64 Å (C-O). Also, the binding energy is −0.236 eV, which is relatively higher than the interaction energy of DNT/epoxy—1. In Figure 4c (DNT/epoxy—3), the epoxy is aligned parallel to the DNT with a minimum distance of 3.60 Å (C-O) and a binding energy of −0.168 eV, which is the lowest among the three configurations of DNT/epoxy composites.

**Figure 5:**
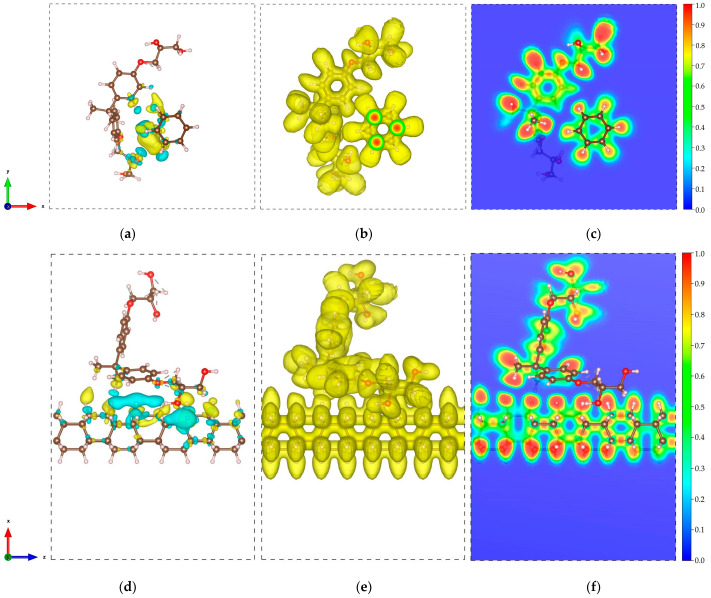

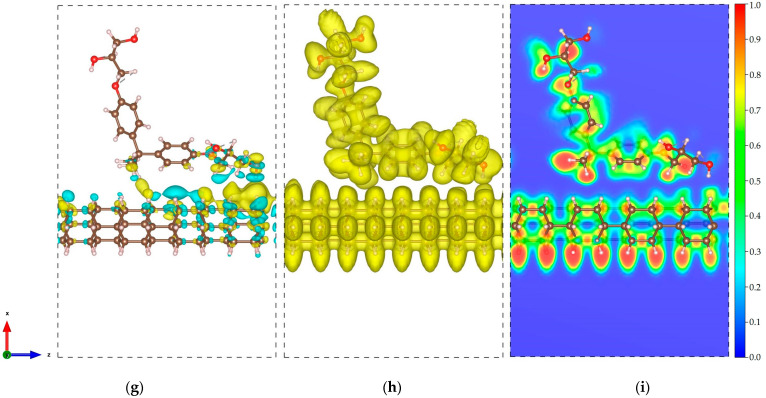
Electronic visualization of the DNT/epoxy composites: (**a**,**d**,**g**) charge density difference (CDD) (0.0002 e/Å^3^); (**b**,**e**,**h**) ELF isosurface (0.4); (**c**,**f**,**i**) 2D ELF contour of (**a**–**c**) DNT/epoxy—1, (**d**–**f**) DNT/epoxy—2, and (**g**–**i**) DNT/epoxy—3. The yellow and cyan isosurfaces denote the accumulation and depletion of the electronic charge, respectively.

In all configurations, the C-C and C-H bond lengths of the DNTs are 1.54–1.58 Å and 1.10 Å, with bond angles of 106.2–106.7°. On one hand, the C-C, C-H, C-O, and O-H bond lengths of the epoxy are measured to be 1.39–1.54 Å, 1.09–1.11 Å, 1.37–1.43 Å, and 0.96–0.97 Å, respectively, with bond angles of 117.1–122.3°. These results also show minor deviations in the structures of the subsystems, which indicates minimal interaction between the DNT and epoxy. Similar to the discussions above, all structures display noncovalent interactions and the negative values of binding energies indicate an exothermic reaction. The non-bonded interactions between the DNT and epoxy, as well as the measured minimum interaction distances, demonstrate physisorption mainly governed by weak vdW forces. The CDD and ELF calculations are also performed for DNT/epoxy composites and are presented in Figure 5. 

Our results show similar behavior to that of DNT/cellulose composites. The CDD isosurface shows that the accumulation and depletion of charges is mainly distributed along the nanothread interface. Bader charge transfer analysis reveals 0.003 e (DNT/epoxy—1) and 0.019 e (DNT/epoxy—2) charge transfer from the epoxy to the nanothread. A 0.002 e charge transfer from the DNT to epoxy is obtained for the DNT/epoxy—3 composite. The DNT/epoxy complex systems’ ELF isosurface shows uniform electron density across the composites. The 2D ELF plots confirm the non-bonded interactions with the visible blue region between the epoxy and nanothread. The ELF plots further highlight the non-bonding interactions between the nanothread and epoxy. 

Owing to the hydrogenated surface of DNT, the vdW interactions between the polymer and nanothread are weaker than those in similar systems. This behavior is consistent with the previously reported interactions of diamond nanothreads with other polymers [33,34,35,36,75,76,77].

**Figure 6:**
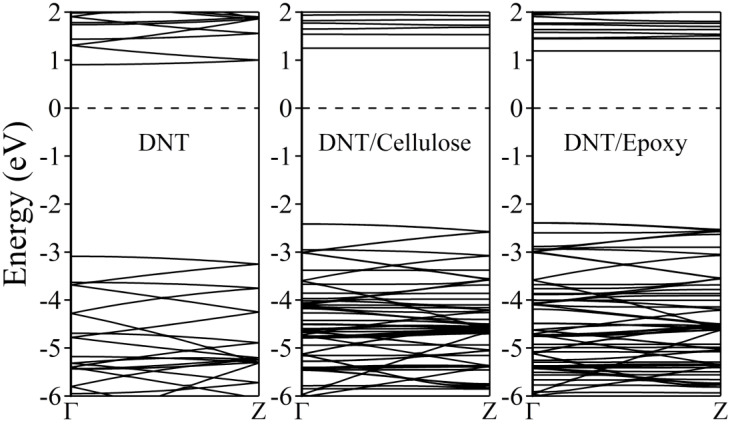
Electronic band structures of (3,0) DNT and the DNT/cellulose−1 and DNT/epoxy−1 composites. The Fermi level is set to zero and is designated by the horizontal dashed line at 0 eV.

Figure 6 illustrates the calculated electronic band structure of (3,0) tube DNT. According to the GGA/PBE DFT calculations, benzene-derived diamond nanothreads are insulators with at least a 3.5 eV energy band gap, which is expected due to the intrinsic sp^3^ bonding of carbon atoms just like diamond [29,30,78,79,80,81,82]. Our result shows that pure sp^3^-(3,0) DNT has a direct band gap of 3.99 eV at the Γ point, which is close to the previously calculated value (3.98 eV) [82]. The complex systems, meanwhile, show a slightly lower band gap. We observe a shift of bands at the higher energy level, resulting in the DNT/cellulose—1 and DNT/epoxy—1 composites having direct band gaps of ~3.7 eV and ~3.6 eV, respectively. These minor modifications in the energy band gap are attributed to the weak interaction between the nanothread and polymers and the minimal charge transfer previously mentioned. This, in turn, leads to the realignment of the energy states. As discussed above, the interaction energies of the DNT/cellulose—1 and DNT/epoxy—1 composites are 0.797 eV and 0.224 eV, respectively. The charge transfer from the cellulose to the nanothread is calculated to be 0.008 e, while the charge transfer from the epoxy to the DNT is 0.003 e. However, it is also noteworthy that GGA usually underestimates the band gap of materials.

**Figure 7:**
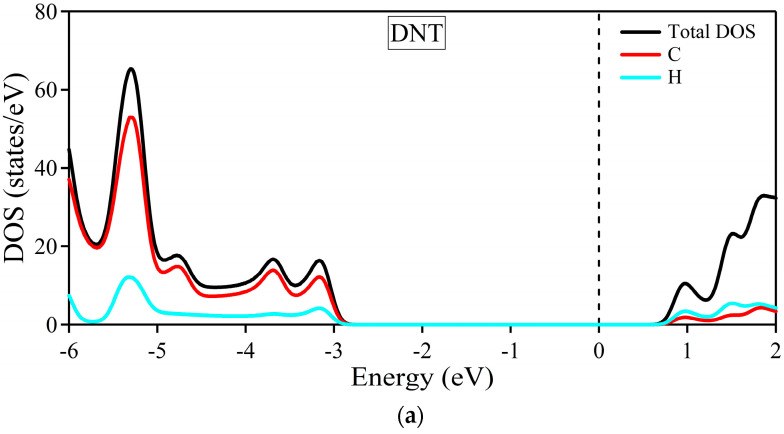

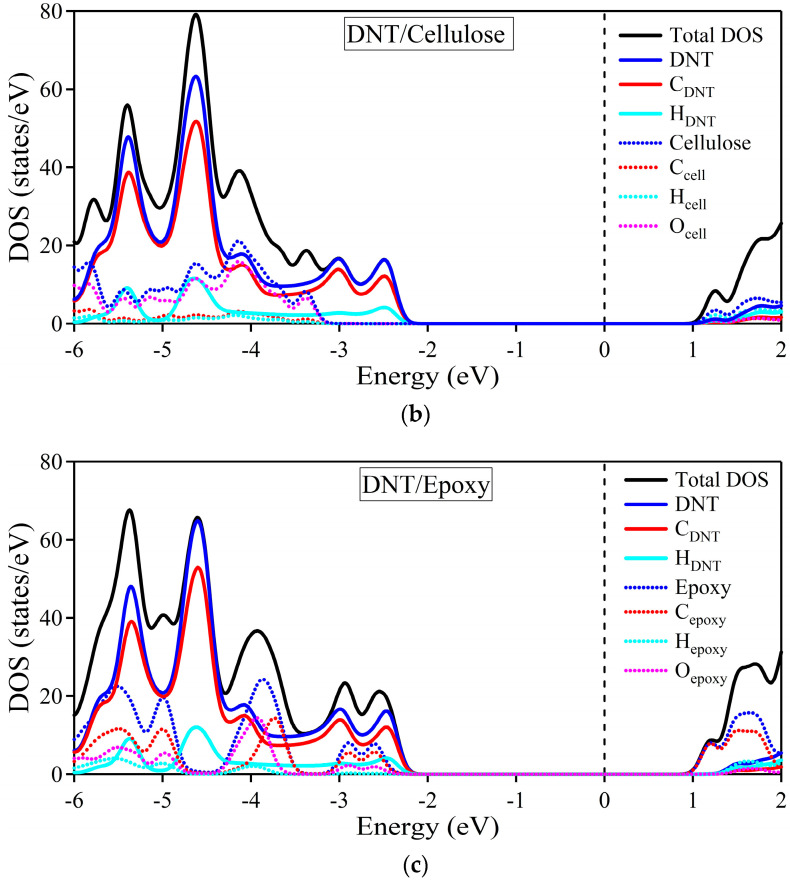
Total and partial density of states of (**a**) (3,0) DNT, (**b**) DNT/cellulose−1, and (**c**) DNT/epoxy−1. The Fermi level is set to zero and is designated by the vertical dashed line at 0 eV.

To further inspect the composites’ electronic band structures, the total and partial density of states are provided in Figure 7. The black solid line depicts the total DOS, while the DNT and cellulose/epoxy contributions are represented by blue solid and dotted lines, respectively. The DOS of the carbon and hydrogen atoms of DNT are outlined by red and cyan solid lines, respectively. Also, the carbon, hydrogen, and oxygen atoms of the cellulose/epoxy are depicted by red, cyan, and magenta dotted lines, respectively. 

In Figure 7a, the density of states of the DNT/cellulose composite shows that at the lower energy range [~−2.3 eV, −6 eV], most contribution to the total DOS of the composite, comes from DNT, specifically from the carbon atoms of the nanothread. The cellulose shows a significant contribution at ~−3.1 eV to −6 eV, with its oxygen atoms having the highest DOS relative to the total contribution of the cellulose. Similarly, the DOS of the DNT/epoxy nanocomposite presented in Figure 7b shows that most contribution to the total DOS still comes from the DNT, specifically from its carbon atoms. The contributions of the epoxy in the valence band are observed at −2.5 eV to −3.0 eV, −3.5 eV to 4.5 eV, and −4.8 eV to −6.0 eV, mostly coming from its carbon and oxygen atoms. At around −3.5 eV to −4.5 eV, higher contributions of the atoms from epoxy are observed compared to those of DNT.

Overall, it can be noticed that in both complex systems the DOS of the composites is practically a superposition of the DOS of their respective subsystems. Moreover, we notice no strong hybridization between atoms of the nanothread and polymer. The primary features of the DNT are reproduced, suggesting that the electronic properties of the nanothread are preserved. These observations further support our claim that DNT and cellulose/epoxy only interact weakly. Our results generally reveal minimal alterations in the electronic structures of the DNT when interacting with polymer (cellulose/epoxy), which shows good agreement with previous theoretical studies on related systems (nanomaterial/polymer composites) [47,83,84,85,86].

**Figure 8:**
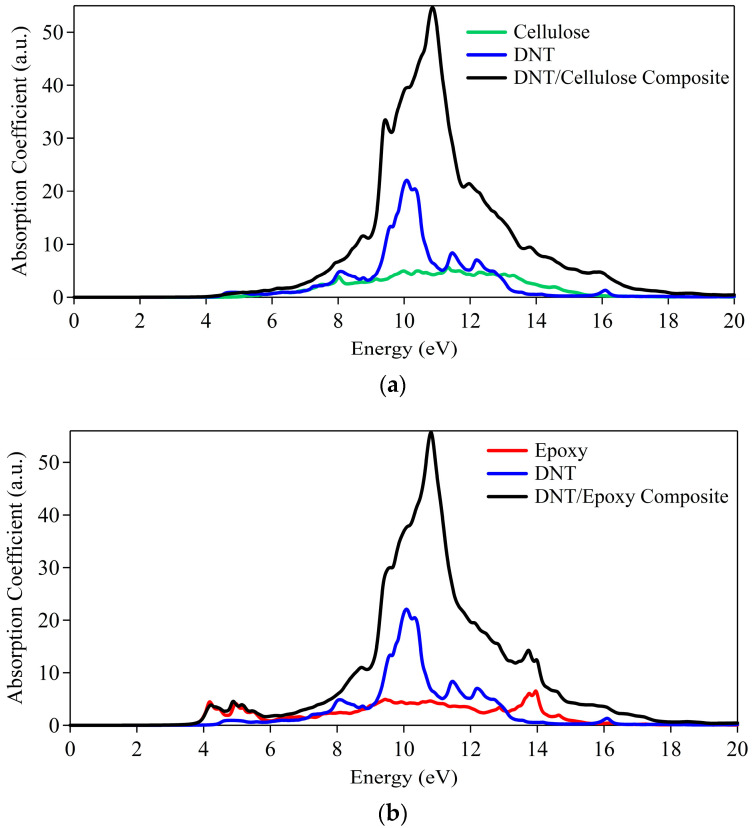
Absorption spectra of (**a**) DNT/cellulose and (**b**) DNT/epoxy composites.

We have also explored the optical absorption spectra of the DNT/cellulose and DNT/epoxy composites, as displayed in Figure 8. These spectra offer information on how these composites exhibit absorption features due to their electronic structure. The onset of absorption where the absorption coefficient starts to rise from zero corresponds to the energy required to excite an electron from the valence band to the conduction band in semiconducting and insulating materials.

Illustrated in Figure 8a are the absorption spectra of the DNT/cellulose composite, which show that the absorption of DNT onsets at ~4.4 eV and that of cellulose onsets at ~5.2 eV, indicating their insulating behavior (i.e., wide bandgap). Moreover, the combined system reproduces the same features as the cellulose and the DNT. The absorption spectra of DNT/epoxy presented in Figure 8b reveal that the epoxy has absorption starting at ~3.6 eV while the DNT appears at ~4.4 eV, similar to the observation made in Figure 8a. Likewise, the absorption spectrum of the DNT/epoxy composite displays the corresponding absorption features of its subsystems. Overall, the results show minor alterations in the absorption spectra of the nanothread, implying that the optical property of the diamond nanothread is maintained. Furthermore, these results reinforce the large energy band gaps revealed on the electronic structures of the composites.

### 2.2. Calcium Silicate Hydrate and Boron Nitride Nanotube Nanocomposites

**Figure 9:**
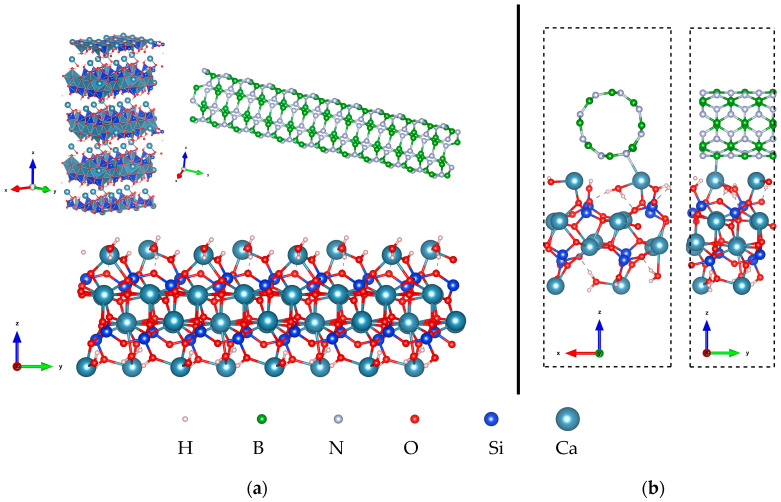
(**a**) Crystal structure of the C-S-H model, single-layer C-S-H, and BNNT(4,4). (**b**) Optimized configuration of the C-S-H/BNNT(4,4) nanocomposite showing the unit cell.

The optimized atomic structure of the C-S-H model, single-layer C-S-H, BNNT(4,4), and the optimized configuration of the C-S-H/BNNT(4,4) composite are presented in Figure 9. Herein, the C-S-H/BNNT composite was modeled with compatible lattice constants to ensure feasible simulations. To simulate the interface of the C-S-H with the BNNT, a single layer of the C-S-H taken from the molecular dynamics results is utilized [87]. The intralayer of prototype C-S-H has a 1:1 Ca to Si ratio, while two Ca ions and two water molecules are placed at the surfaces of the C-S-H. This model parallels the recent research showing that the surface of C-S-H is rich in Ca ions [88]. Both the tendency of the Ca ions to diffuse in the system and the variability of the Ca:Si ratio are probable [64]. These situations will not be covered in this work.

The prototype C-S-H here is adapted to our recent work that describes the interface of graphene nanoribbon and SWCNT with the C-S-H [89]. The isolated C-S-H has Ca–H_2_O, Ca–O, and Si–O bonds ranging from 2.35 to 2.43 Å, 2.13 to 2.70 Å, and 1.60 to 1.72 Å, respectively. The bond lengths are in agreement with experimental data [88]. Turning to the isolated BNNT, the B-N bond in the tube direction is measured to be 1.44 Å, while in the circumferential direction the B-N bond is 1.45 Å; their bond angles range from 116.0° to 120.4°. The measured bond lengths here are close to the BNNT report [90]. The deviation is attributed to the fixed unit cell considered here and the overall method limitation.

The minimum interaction distance of C-S-H and BNNT is 2.68 Å (Ca–N). The interaction distance of the H_2_O—BNNT is 3.10 Å (O–B), and the Si-OH—BNNT distance is 2.40 Å and 3.21 Å for H-N and O-N, respectively. In the composite, the C-S-H has Ca–H_2_O, Ca–O, and Si–O bonds ranging from 2.34 to 2.73 Å, 2.10 to 2.62 Å, and 1.62 to 1.72 Å, respectively. Meanwhile, the B-N bond of the BNNT along the tube and circumferential directions are measured to be 1.44–1.47 Å and 1.44–1.46 Å, with the bond angles of 112.9° to 121.4°. The most observable change in the atomic structure of the C-S-H in the complex structure is the orientation of the confined H_2_O, in which the H points toward the O atoms, establishing a hydrogen bond. These minor structural changes are mainly signals of weakly bonded systems similar to the discussion in the preceding section. The interaction strength is quantified by calculating the binding energy per unit cell. The binding energy of the C-S-H/BNNT is −0.89 eV, which is lower in magnitude than the binding energy of the C-S-H/SWCNT (−5.0 eV) and C-S-H/graphene nanoribbon (−3.24 eV) [89]. Furthermore, the calculated binding energy here is much lower than the binding energy of a graphene-based material with the monomer of C-S-H [91].

**Figure 10:**
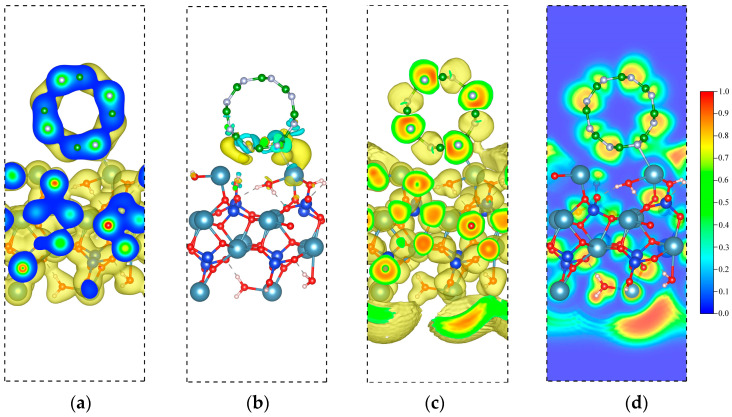
Electronic visualization of the C-S-H/BNNT (4,4) nanocomposite: (**a**) pseudo-charge density (0.02 e/Å^3^); (**b**) charge density difference (CDD) (10^−3^ e/Å^3^); (**c**) ELF isosurface (0.4); (**d**) 2D ELF contour.

The CDD and ELF are also provided to understand the interaction of the BNNT and C-S-H composite. At the isosurface value of 0.02/Å^3^, it is noticeable that the Ca and N bonds overlapped, indicative of a shared-electron-type interaction. We further confirm this result by calculating the CDD isosurface, showing an accumulation of electronic charge at the bond region. The overall charge transfer from C-S-H to the BNNT is 0.47 e. The total electron depletion of the Ca ions at the interface is 0.37 e. The total contribution of the Si-OH and H_2_O is ~0.10 e, which is much lower owing to the size of the system considered. These results indicate that major charge transfer is attributed to the Ca ions at the interface, which drives the interaction of nanocomposites. This further confirms the findings in our recent work on the Ca ions at the interface, which drives the bonding between the C-S-H layer and the graphene nanoribbon and SWCNT. The ELF isosurface and 2D contour are displayed in Figure 10c and Figure 10d, respectively, to reinforce our claims. The result shows that the BNNT tends to interact with the lone pair of the Ca ions of the C-S-H. This is contrary to our previous work, where the Ca ion’s lone pair vanishes upon adsorption of the graphene nanoribbon and SWCNT.

**Figure 11:**
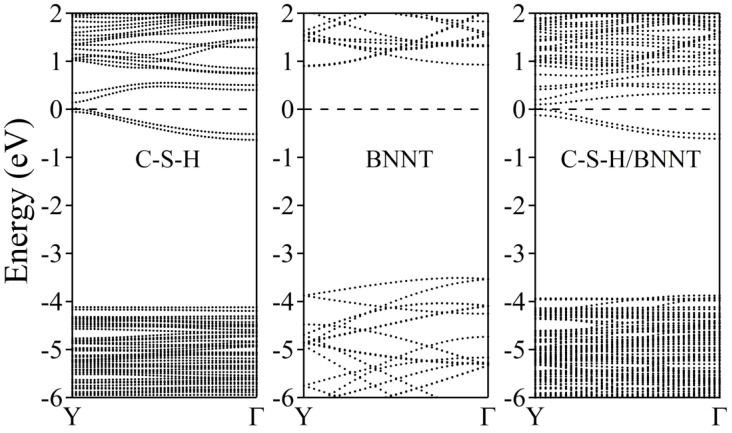
Electronic band structure of C-S-H, BNNT (5,5), and the C-S-H/BNNT complex. The Fermi level is set to zero and is designated by the horizontal dashed line at 0 eV.

**Figure 12:**
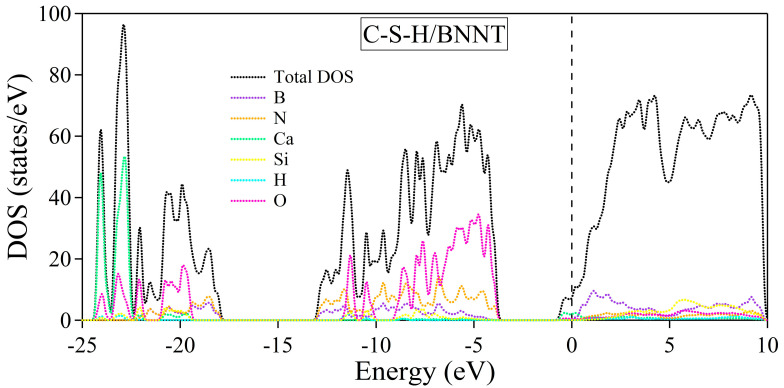
Total and partial density of states of the C-S-H/BNNT complex. The Fermi level is set to zero and is designated by the vertical dashed line at 0 eV.

The electronic band structures of the C-S-H, BNNT, and C-S-H/BNNT complexes are further calculated to reveal the composites’ metallicity, as shown in Figure 11. Our results show that the dispersive bands appear near the Fermi energy due to the lone pair of the Ca ions, as discussed in [89]. The BNNT shows insulating properties, having a bandgap of 3.77 eV located at the Γ point. This is in agreement with the reports presented in ref. [92], with bandgaps of 3.87 eV and 4.11 eV calculated using PBE and BLYP functionals, respectively. The PBE functional is well known for underestimating the bandgap of semiconductors and insulators. Moreover, the theoretical length of the BNNT (3.695 Å) is used here to match the size of the unit cell of the C-S-H, while ref. [92], allowing cell relaxation, gives 2.560 Å, leading to minor disagreement with the bandgap of BNNT, aside from intrinsic error of the theory and approximations combined. The composite electronic band structure can be considered superimposed on the constituents, except for the minor shift of bands of the BNNT to lower energy due to charge accumulations. This is the usual result of weakly bonded systems, as demonstrated in the following references [83,84,85,86,93]. We further provide the system’s total and partial density of states, as shown in Figure 12. The black dotted line depicts the total DOS, while the total contributions of the B, N, Ca, Si, H, and O atoms are depicted by purple, orange, green, yellow, cyan, and magenta dotted lines, respectively. The decomposed DOS of BNNT and C-S-H shows no strong hybridization indicative of weakly bonded systems, confirming our previous conclusions.

**Figure 13:**
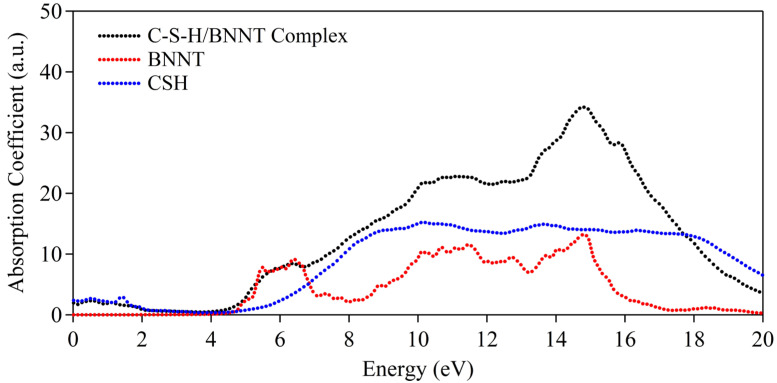
Absorption spectra of C-S-H/BNNT complex.

The absorption spectra are further calculated to provide reference to observables. Here, as can be observed in Figure 13, the BNNT tends to have abrupt absorption at ~4.5 eV, indicative of insulating properties (i.e., wide bandgap nature). Meanwhile, the single C-S-H layer shows metallic characteristics, having minor absorption at the energy region < 3.0 eV. This is attributed to the dispersive bands at the vicinity of the Fermi level. In the complex structure, both the characteristics of BNNT and C-S-H absorption spectra are reproduced except for a minor deviation owing to the realignment of bands. It should be noted that in a bulk system of C-S-H, the dispersive bands may vanish due to the presence of bulk H_2_O in the interface. On the whole, the absorption features of the boron nitride nanotube are preserved. Moreover, these results provide additional confirmation of the electronic behavior of the C-S-H/BNNT composite and its subsystems.

## 3. Materials and Methods

The DFT calculations are performed using the Quantum ESPRESSO v.6.4.1 package [94,95]. In all calculations, the optimized norm-conserving pseudopotential [96], generalized gradient approximation (GGA) within Perdew–Burke–Ernzerhof parametrization [97], and plane-wave basis sets are used. The energy and charge density cutoff are set at 60 Ry and 240 Ry, respectively. The energy and force tolerance are set to $10^{-8}$ Ry and $10^{-4}$ Ry/Bohr. The standard semi-empirical Grimme-D3 [98] van der Waals (vdW) correction is utilized to take into account the weak interaction between subsystems.

Gamma-point-specific algorithms are used for the structural optimization calculations on DNT/polymer composites. The well-established sp^3^ tube (3,0) nanothread (corresponding to 123456 structure [29]) is utilized in our calculations. This DNT type was theoretically proposed [99] even before the experimental demonstration of nanothreads. It is also classified as the lowest energy achiral nanothread [29] and has a uniform and regular structure. The DNT/cellulose composites are modeled by considering three orientations of cellulose with respect to the nanothread and are labeled as DNT/cellulose—1, DNT/cellulose—2, and DNT/cellulose—3. The dimension of the simulation box is 20.00 Å × 20.00 Å × 17.34 Å. Similarly, three orientations of epoxy with respect to the DNT are constructed and labeled as DNT/epoxy—1, DNT/epoxy—2, and DNT/epoxy—3. The simulation boxes for each of the DNT/epoxy composites have the dimensions 22.00 Å × 22.00 Å × 17.34 Å (DNT/epoxy—1), 26.00 Å × 22.00 Å × 17.34 Å (DNT/epoxy—2), and 28.00 Å × 20.00 Å × 17.34 Å (DNT/epoxy—3). For the C-S-H/BNNT complex system, a 3 × 4 × 1 Monkhorst-Pack grid [100] is used for the optimization calculation, and the dimensions of the simulation box are 11.16 Å × 7.39 Å × 30.00 Å.

To examine the stability of the composites, binding energy ($E_{b}$) is also calculated using the expression:

(1) $$E_{b}=E_{Composite}-\sum E_{Subsystems}$$

where $E_{Composite}$ and $E_{Subsystems}$ denotes the total energy of the combined structure and isolated subsystems, respectively. The charge density distribution (CDD) and electron localization function (ELF) are also obtained for all structures to visualize the electron redistributions. Both provide descriptive insights into the redistribution of the electronic distribution and are thus essential in distinguishing the nature of bonds in the complex structure, as demonstrated in our previous works on similar systems [83,84,89]. The CDD and ELF images are visualized using VESTA v.3 [101]. Bader charge transfer analysis [102,103,104,105], which implements the zero-flux approach in dividing atoms in many-body electronic systems, is performed, and the charge transfer is calculated using the expression:

(2) $$\Delta \rho =\rho_{composite}-\rho_{isolated}$$

where $\rho_{composite}$ and $\rho_{isolated}$ are the charges of the atoms of interest in the composite and isolated systems. Refined 1 × 1 × 29 and 10 × 14 × 1 Monkhorst–Pack grids are utilized for the electronic structure and optical property calculations of the DNT/polymer and C-S-H/BNNT systems, respectively.

## 4. Conclusions

We have studied the interactions, electronic structures, and optical properties of DNT/cellulose, DNT/epoxy, and C-S-H/BNNT composites through density functional theory calculations. Various orientations of DNT/cellulose and DNT/epoxy composites have been considered. Our results reveal wide minimum interaction distances between non-hydrogen atoms of the nanothread and polymers, less binding energies, and minimal charge transfer, indicating physical adsorption and weakly interacting systems mainly of the vdW type, as further discerned from the CDD and ELF plots. Consequently, only minor modifications to the electronic properties of the nanothread occurred. The electronic band structures of DNT/polymer systems show a slight shift of bands at the higher energy level relative to that of DNT. The density of states displays no hybridization between atoms of the nanothread and polymer, confirming their weak interactions. The optical absorption spectra of the composites indicate insulating behavior.

On the other hand, the BNNT and C-S-H composite shows a shared type of interaction, as indicated by the formed Ca-N bond and accumulation of electronic charge at the bond region. Bader charge transfer analysis further confirms that most charge transfer from the C-S-H to the BNNT comes from the Ca ions. The electronic band structure of the complex system shows superimposed bands of BNNT and C-S-H. The density of states reveals no hybridization, which further confirms the weak interaction between the subsystems. The absorption spectra of the C-S-H/BNNT complex show metallic characteristics.

Overall, this work provides fundamental information on the bonding characteristics and the electronic and optical properties of the DNT/polymer (cellulose and epoxy) and BNNT/C-S-H composites, which are otherwise not readily known in experiments. This information is useful in giving experimentalists an electronic-level perspective for tuning the properties of nanomaterial-reinforced composites for targeted applications.

## Figures and Tables

**Table 1:** Calculated minimum distances ($d_{min}$), binding energies ($E_{b}$), and charge transfers (Δρ) of the DNT/cellulose composites.

System	$d_{min}$ (Å)	$E_{b}$ (eV)	Δρ (e)
DNT/cellulose—1	3.67	−0.797	−0.008
DNT/cellulose—2	3.42	−0.730	0.004
DNT/cellulose—3	3.48	−0.726	−0.006

**Table 2:** Calculated minimum distances ($d_{min}$), binding energies ($E_{b}$), and charge transfers (∆ρ) of the DNT/epoxy composites.

System	$d_{min}$ (Å)	$E_{b}$ (eV)	Δρ (e)
DNT/Epoxy—1	3.56	−0.224	−0.003
DNT/Epoxy—2	3.64	−0.236	−0.019
DNT/Epoxy—3	3.60	−0.168	0.002

## Data Availability

Data are contained within the article.
